# Does COVID-19 Infection Increase the Risk of Diabetes? Current Evidence

**DOI:** 10.1007/s11892-023-01515-1

**Published:** 2023-06-07

**Authors:** Rachel Wong, Emily Lam, Carolyn T. Bramante, Steven G. Johnson, Jane Reusch, Kenneth J. Wilkins, Hsin-Chieh Yeh

**Affiliations:** 1 Department of Biomedical Informatics, Stony Brook University, Stony Brook, NY USA; 2 Health Science Center, Stony Brook Medical Center, Level 3, Room 45101 Nicolls Road, Stony Brook, NY 11794 USA; 3 Division of General Internal Medicine, University of Minnesota Medical School, Minneapolis, MN USA; 4 Institute for Health Informatics, University of Minnesota, Minneapolis, MN USA; 5 Division of Endocrinology, Metabolism & Diabetes, University of Colorado Anschutz Medical Campus, Aurora, CO 80045 USA; 6 Biostatistics Program/Office of Clinical Research Support, National Institute of Diabetes and Digestive and Kidney Diseases, National Institutes of Health, Bethesda, MD USA; 7 Department of Medicine, Johns Hopkins University, Baltimore, MD USA; 8 Department of Epidemiology, Johns Hopkins University, Baltimore, MD USA

**Keywords:** Incident diabetes, SARS-CoV-2, COVID-19, Type 1 diabetes, Type 2 diabetes

## Abstract

**Purpose of Review:**

Multiple studies report an increased incidence of diabetes following SARS-CoV-2 infection. Given the potential increased global burden of diabetes, understanding the effect of SARS-CoV-2 in the epidemiology of diabetes is important. Our aim was to review the evidence pertaining to the risk of incident diabetes after COVID-19 infection.

**Recent Findings:**

Incident diabetes risk increased by approximately 60% compared to patients without SARS-CoV-2 infection. Risk also increased compared to non-COVID-19 respiratory infections, suggesting SARS-CoV-2-mediated mechanisms rather than general morbidity after respiratory illness. Evidence is mixed regarding the association between SARS-CoV-2 infection and T1D. SARS-CoV-2 infection is associated with an elevated risk of T2D, but it is unclear whether the incident diabetes is persistent over time or differs in severity over time.

**Summary:**

SARS-CoV-2 infection is associated with an increased risk of incident diabetes. Future studies should evaluate vaccination, viral variant, and patient- and treatment-related factors that influence risk.

## Introduction

As of April 2023, there have been over 762 million confirmed cases of COVID-19 with over 6.8 million deaths globally [1]. Certain comorbidities, including diabetes mellitus, have high prevalence in patients with COVID-19 with reports ranging from 5.3 to 35.5% [2, 3]. Similar to other infectious diseases, individuals with diabetes and poor glycemic control are at increased risk for poor outcomes in acute COVID-19 [4, 5]. Furthermore, the observation of an increase in newly diagnosed diabetes after acute COVID-19 infection suggests a bidirectional relationship between the SARS-CoV-2 and diabetes [6–8]. Given the already high global prevalence of diabetes, and the impact of the disease on human morbidity, mortality, quality of life, and health expenditures [9], it is important to understand the impact of COVID-19 on the global epidemiology of diabetes. In this review, we consider the evidence pertaining to the risk of incident diabetes, including type 1 and type 2 subtypes, after COVID-19 infection.

## Proposed Mechanisms for Incident Diabetes After SARS-CoV-2 Infection

Multiple mechanisms have been proposed to contribute to the pathophysiology of incident diabetes after SARS-CoV-2 infection. Studies showing preferential ACE2 expression in human pancreatic tissue highlight the potential for a direct effect of the virus on β-cells and the pancreatic microvasculature or ductal cells [10–14]. Increased inflammation and activation of cytokines during acute infection may also lead to damage to β-cells and reduced insulin secretion and acute insulinopenia [8, 11, 15, 16], with concomitant cytokine-mediated insulin resistance in the liver and skeletal muscle important for insulin-mediated disposal of glucose [17]. In addition to the relative insulin deficiency associated with SARS-CoV-2 infection, new insulin resistance may also play an important role in hyperglycemia during acute infection [18–20] and has been reported within 6 months of follow-up in patients without pre-existing diabetes [21, 22]. Non-pancreatic mechanisms, such as elevation of hormones like GP73 in SARS-CoV-2 infection, have also been proposed as mechanisms for hyperglycemia through increased hepatic glucose production [23, 24]. Iatrogenic hyperglycemia from medications such as corticosteroids may also contribute to the development of new diabetes in susceptible patients [8, 25, 26]. Many of these physiological and iatrogenic stressors are transient and some reports suggest that stress hyperglycemia may be transient and that a proportion of patients regress to normoglycemia [22, 27, 28]. Viral respiratory infections increase the risk of autoimmune diabetes [29] and mechanisms such as antigen presentation, molecular mimicry, and hyperinflammatory states associated with cytokine storm from SARS-CoV-2 infection have also been proposed in triggering increased incidence of type 1 diabetes [30–33].

In addition to the proposed pathophysiologic mechanisms related to the rise in diabetes diagnoses, social and environmental factors surrounding the pandemic may also have contributed to increased incident diabetes. During the pandemic, physical activity declined significantly and rates of overweight and obesity increased; patients also deferred routine healthcare which could affect behavioral modification and risk reduction [34–39], contributing to insulin resistance and the development of diabetes. Acute SARS-CoV-2 infection may have brought new patients into the healthcare system who may have had previous undiagnosed diabetes. A recent population-level assessment of the temporal relationship between SARS-CoV-2 and new diabetes diagnoses showed a sharp increase in incident diabetes cases at the time of SARS-CoV-2 infection, followed by a decrease in new diabetes diagnoses in the months after infection. These findings suggest that patients with pre-existing diabetes could be simply diagnosed because of new interaction with the healthcare system, or that the metabolic challenge of SARS-CoV-2 infection could cause earlier presentation of diabetes and early diagnosis of at-risk individuals [40]. There is also evidence that incident diabetes is associated with younger age, Hispanic and non-White ethnicity and race, and Medicaid insurance, suggesting a sociodemographic pattern of increased diagnosis in patients with decreased access to care [28].

## Studies Evaluating Incident Diabetes After SARS-CoV-2 Infection

Multiple studies report significantly higher incidence of diabetes in patients following acute SARS-CoV-2 infection, both in the primary (Table 1) and secondary (Table 2) literature. The studies were conducted using electronic health records, claims, or registry databases in US, Canadian, and European populations. The majority of studies included in this review were recent meta-analyses or retrospective observational studies that utilized two main matched comparator groups: (1) historical and contemporary controls without SARS-CoV-2 infection and (2) controls with acute respiratory infection, viral respiratory infection, pneumonia, or influenza. Most studies defined incident diabetes using ICD codes for diagnosis, with only 3 studies also including laboratory-based measurements of HbA1c > 6.5%, fasting glucose level > 126 mg/dL, or random glucose level > 200 mg/dL in their definition [42, 43, 48]. Covariates that were included in propensity score matching differ widely between studies and there was a lack of standardization in the duration of follow-up after SARS-CoV-2 infection. There was also variability in analyses and reported estimated risk; most reported odds ratios (OR), while some others reported relative risk (risk ratios, RR) and hazard ratios (HR).

**Table 1:**
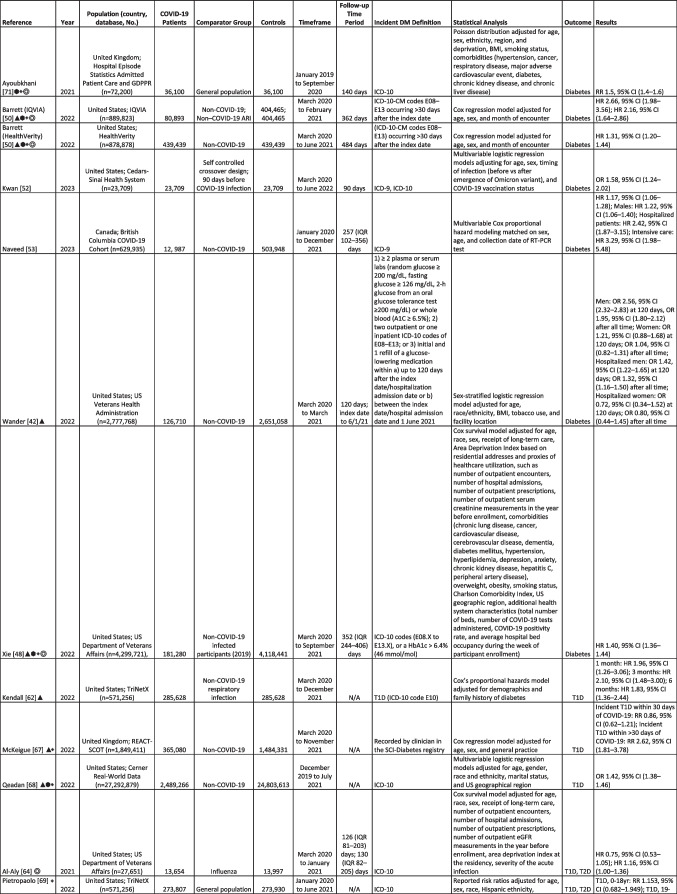

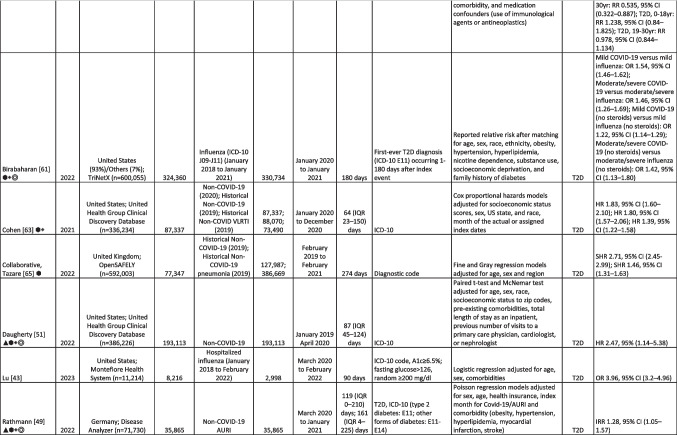
Characteristics of included cohort studies reporting COVID-19 and risk of diabetes

*AURI*, acute respiratory tract infection; *VLRTI*, viral lower respiratory tract illness; *ARI*, acute respiratory infection; *ICD-10*, International Statistical Classification of Diseases and Related Health Problems–Tenth Revision; *ICD*-*9*, International Statistical Classification of Diseases and Related Health Problems–Ninth Revision; *IQR*, interquartile range; *T1D*, Type 1 Diabetes; *T2D*, Type 2 Diabetes; *OR*, odds ratio; *RR*, relative risk; *HR*, hazard ratio; *IRR*, incidence rate ratio; *SHR*, sub distribution hazard ratio; Included in the following meta-analyses: ▲, Ssentongo,  , Zhang, ◈, Lai, ◎, Banerjee

**Table 2:** Characteristics of included meta-analyses reporting COVID-19 and risk of diabetes

Reference	Year	Included studies/cohorts	Timeframe	Comparator group	Statistical analysis	Outcome
Banerjee [44.••••]	2022	4 studies (5 datasets) post-COVID-19 versus matched controls (*n* = 5.8 million); 3 studies, COVID-19 versus severity matched influenza in mild (*n* = 308,613) and moderate-severe/hospitalized (*n* = 24,090)	Database inceptions to April 2022	Non-COVID-19, influenza (severity matched)	Generic inverse variance method using the fixed-effects/random-effects model with heterogeneity was measured using *I*^2^ statistics	HR 1.59, 95% CI (1.40—1.81); Moderate-severe/hospitalized cases: HR 1.52, 95% CI (1.36–1.70); Mild cases: HR 1.22, 95% CI (1.14—1.31)
Lai [45.••••]	2022	10 studies (11 datasets) (*n* = 47.1 million)	Database inceptions to June 2022	Non-COVID-19	Random effects model and DerSimonian and Laird method with heterogeneity evaluated by Cochran’s *Q*-test	Diabetes: RR 1.64, 95% CI (1.51—1.79); T2D: RR 1.78, 95% CI (1.56—2.02); T1D: RR 1.42, 95% CI (1.38—1.46)
Ssentongo [46.••••]	2022	8 studies (*n* = 47.4 million); (*n* = 4.2 million) COVID-19 patients and (*n* = 43.2 million) controls	December 2019 to October 2022	Non-COVID-19	Random-effects meta-analyses using the generic inverse variance method and Duval and Tweedie’s trim and fill test with heterogeneity was estimated by DerSimonian and Laird random-effects method	Pooled point estimate 1.66, 95% CI (1.38–2.00), United States: pooled point estimate 1.77, 95% CI (1.41–2.22), Europe: pooled point estimate 1.33, 95% CI (1.14–1.56)
Zhang [47.••••]	2022	9 studies (*n* = 40 million)	Database inceptions to June 2022	Non-COVID-19, Non-COVID-19 AURI	Freeman–Tukey double arcsine method, inverse variance random-effects meta-analysis (DerSimonian and Laird) with heterogeneity assessed with the Cochran *Q* and *I*^2^ statistics	Diabetes: RR 1.82, 95% CI (1.47–2.24), T1D: RR 1.48, 95% CI (1.26–1.75), T2D: RR 1.70, 95% CI (1.32–2.19), Unspecified type of diabetes: RR 1.50, 95% CI (0.87–2.58); diabetes: RR 1.17, 95% CI (1.02–1.34)

### Risk of Incident Diabetes Compared to Non-COVID-19-Infected Controls

The current literature includes several large meta-analyses that report similar risk estimates for incident diabetes compared to matched controls without SARS-CoV-2 infection (Table 2). Banerjee et al. studied 5.8 million patients from 5 cohorts and estimated a hazard ratio of 1.59 (95% CI 1.40–1.81) for incident diabetes after SARS-CoV-2 infection [44.••••]. Lai et al. conducted a meta-analysis that included 11 retrospective cohorts from the United States (US), Europe and the global population with 47.1 million participants and calculated a 64% increased risk of overall diabetes (RR 1.64, 95% CI 1.51–1.79). They did not detect any significant differences between age, regions, races, or length of follow-up, but pooled risk estimates from 2 studies showed a potentially greater risk in males compared to females (RR 1.45, CI% 1.37–1.53) [45.••••]. Ssentongo et al. reviewed 8 studies from 3 different countries that included 47.4 million overall and 4.3 million COVID-19 patients and used a random effects model to calculate a pooled point estimate of 1.66 (95% CI 1.38–2.00) for increased risk of incident diabetes. They also reported a higher risk in studies from the US of 1.77 (95% CI 1.41–2.22) than Europe 1.33 (95% CI 1.14–1.56) [46.••••]. Zhang et al. found the overall incidence of diabetes to be 15.53 cases per 1000 person-years of follow-up and increased risk (RR 1.62, 95% CI 1.45–1.80) among 9 studies with 34.7 million patients and 4 million patients with COVID-19. They found significant risk increases in all age groups and in both genders, and that risk was highest at less than 3 months from SARS-CoV-2 infection (RR 1.95, 95% CI 1.85–2.06) and with severe infection (RR 1.67, 95% CI 1.25–2.23). They also calculated the *E*-value, or the minimum strengths of associations from uncontrolled confounders that would be needed jointly with COVID-19 and DM across all studies to shift the results significantly, to be 2.08 [47.••••]. Of note, there was some overlap in the study datasets used to conduct these reviews, with all meta-analyses including patients from four primary study databases which utilized data from the US Veterans Health Administration and claims databases in the US and Germany [48–51].

Primary literature that was not included in the meta-analyses also report elevated risk of incident diabetes after SARS-CoV-2 infection. A study of 23,709 patients with acute SARS-CoV-2 infection in the Cedars-Sinai Health System in California reported an adjusted odds ratio of 1.58 (95% CI 1.24–2.02) of incident diabetes in a self-controlled exposure crossover design that occurred 90 days after vs. before acute SARS-CoV-2 infection compared to new benchmark diagnoses of either urinary tract infection or gastroesophageal reflux. The study also found that diabetes risk was higher in the unvaccinated patients (OR 1.78, 95% CI 1.35–2.37), but no association with age, sex, or timing of infection regarding Omicron variant [52]. A recent study of 629,935 individuals from the British Columbia COVID-19 Cohort reported an adjusted hazard ratio of 1.17 (95% CI 1.06–1.28) of incident diabetes, with higher risk in males (adjusted HR 1.22, 95% CI 1.06–1.40). They also found that risk increased for patients requiring hospitalization (adjusted HR 2.42, 95% CI 1.87–3.15) and intensive care (adjusted HR 3.29, 95% CI 1.98–5.48) [53].

Overall, there have been a number of observational studies that show an increased risk of approximately 60% of incident diabetes after SARS-CoV-2 infection compared to patients without COVID-19 from diverse datasets. Point estimates from studies conducted on populations in the US and Europe were similar and validate the generalizability of findings in these geographic locations. A single study from Canada also reported increased risk, but of a smaller magnitude than previous studies [53].

### Risk of Incident Diabetes Compared to Controls with Non-COVID-19 Respiratory Infection

Acute viral infections are known to be associated with development of incident diabetes, with reports of increased risk of type 1 diabetes (T1D) following influenza and other viral infections [29, 54–57], and of type 2 diabetes (T2D) after viral CMV and hepatitis C infection [58–60]. The use of other respiratory infections as a comparator group is relevant in assessing whether incident diabetes is attributable to SARS-CoV-2-mediated mechanisms rather than general morbidity after respiratory illness, and is more representative of people with similar health-seeking behaviors [61]. Multiple studies assessed non-COVID-19 respiratory infections as a comparator group, including acute respiratory infection (ARI) [49, 50, 62, 63], influenza [43, 61, 64], and pneumonia [65]. Of the two meta-analyses reporting risk of incident diabetes in matched patients with SARS-CoV-2 vs. non-COVID-19 respiratory infections, Zhang et al. reported an overall 1.17-fold (95% CI 1.02–1.34) risk and Banerjee et al. reported an increased risk for mild (HR 1.22, 95% CI 1.14–1.31) and moderate-severe/hospitalized (HR 1.52, 95% CI 1.36–1.70) cases [44.••••, 47.••••]. In addition to studies included in these meta-analyses, Kendall et al. reported an increased risk of T1D in pediatric populations at one month (HR 1.96, 95% CI 1.26–3.06), 3 months (HR 2.10, 95% CI 1.48–3.00) and 6 months (HR 1.83, 95% CI 1.36–2.44) after SARS-CoV-2 infection [62]. In a retrospective study of incident T2D in the Montefiore Health System in New York, there was an increased risk of new diagnosis during hospitalization (adjusted OR 3.96, 95% CI 3.2–4.96) and at 3-month follow-up (adjusted OR 1.24, 95% CI 1.07–1.45) in patients with COVID-19 compared to those with influenza [43].

## Risk of Type 1 and Type 2 Incident Diabetes After SARS-CoV-2 Infection

Given the underlying pathophysiologic differences and variable impacts on public health and healthcare delivery by diabetes type, it is important to separately understand the risks of type 1 and type 2 diabetes mellitus with SARS-CoV-2 infection [66]. Although many studies report on the risk of incident diabetes, several studies focus specifically on either risk of incident T1D [64, 67–69] in general and pediatric populations [62], or on incident T2D [43, 49, 51, 61, 63–65, 69] after SARS-CoV-2 infection.

### Type 1 Diabetes

The analyses of risk for T1D are mixed regarding significance and interpretation of the association with SARS-CoV-2 infection. Meta-analyses that analyzed diabetes by subtype reported an increased risk of T1D (RR 1.42, 95% CI 1.38–1.46; 52 more per 10,000 persons, 95% CI 47–57 more) [45.••••] and RR 1.48 (95% CI 1.26–1.75) [47.••••] compared to non-COVID-19 patients. A study of 571,256 patients under 18 years old using TriNetX database also reported elevated risk of T1D at 1 month (HR 1.96, 95% CI 1.26–3.06), 3 months (HR 2.10, 95% CI 1.48–3.00), and 6 months (HR 1.83, 95% CI 1.36–2.44) after SARS-CoV-2 infection compared to patients with non-COVID-19 upper respiratory infections [62]. Additionally, although they did not specifically report on risk of T1D, a pediatric study using the IQVIA and HealthVerity claims databases for patients < 18, the authors reported that nearly one half of the patients with new diabetes diagnosis in the study had DKA, which was higher than in pre-pandemic reports of incident T1D. They concluded that given the association of increased risk of diabetes after SARS-CoV-2 infection, the increased frequency of DKA was not solely explained by delayed care [50].

Interestingly, another study using the TriNetX database did not find a significant difference over a 15-month period in diagnosis of T1D between patients 0–18 years old without and with COVID-19 (OR 1.153, 95% CI 0.682–1.95); they did however, find a decreased risk in young adults aged 19–30 without COVID-19 compared to those with SARS-CoV-2 infection (OR 0.535, 95% CI 0.322–0.887) [69]. Al-Aly et al. also did not find any significant difference in risk of T1D (HR 0.75, 95% CI 0.53–1.05) [64]. McKeigue et al. found that the rate ratio for T1D was 2.62 (95% CI 1.81–3.78) within 30 days of SARS-CoV-2 infection, but that it was non-significant (RR 0.86, 95% CI 0.62–1.21) at > 30 days. In an additional analysis, they modeled seasonal and calendar patterns of T1D incidence from January 2015 to January 2022 over 56-day sliding time windows time and found no association with SARS-CoV-2 infection > 30 days previously or in patients with age < 16. The authors argued against a causal effect, indicating that the association within 30 days may be attributable to increased detection as evidenced by the increase in negative tests. They also suggested that given the typical lag in symptoms of 25 days before T1D diagnosis, many of the patients who tested for SARS-CoV-2 < 30 days may already have had diabetes at the time of infection, and that the time course of increased incident diabetes predated the most of the cumulative incidence of COVID-19 in the 0–14 age group [67].

### Type 2 Diabetes

Meta-analyses that analyzed diabetes by subtype reported an increased risk of T2D (RR 1.78, 95% CI 1.56–2.02; 1287 more per 10,000 persons, 95% CI 924–1683 more) [45.••••] and RR 1.70 (95% CI 1.32–2.19) [47.••••] when compared to non-COVID-19 patients. In a retrospective study of incident T2D in the Montefiore Health System in New York, there was an increased risk of new diagnosis during hospitalization (adjusted OR 3.96, 95% CI 3.2–4.96) and at 3-month follow-up (adjusted OR 1.24, 95% CI 1.07–1.45) in patients with SARS-CoV-2 infection compared to those with influenza. Interestingly though, of the patients with T2D diagnosed during hospitalization, persistence of T2D in patients seen in follow-up was higher in influenza patients than COVID-19. Also, of patients who were not diagnosed with T2D during hospitalization, rates of T2D diagnosis at 3-month follow-up were not significantly different between COVID-19 and influenza (adjusted OR 0.90, 95% CI 0.64–1.28) [43]. These findings may be suggestive that T2D diagnoses associated with COVID-19 diagnoses may be transient which is in line with findings from observational studies showing high rates of regression to normoglycemia at follow-up [22, 27, 28] and no significant difference in glycemic control between patients with pre-existing T2D after COVID-19 [70]. Additionally, in an analysis excluding patients with mild COVID-19 or influenza who were treated with steroids, Birabaharan et al. found a weaker relative risk of incident T2D (RR 1.54, 95% CI 1.46–1.62 vs. RR 1.22, 95% CI 1.13–1.80) [61].

## Conclusion

The overall risk of incident diabetes after SARS-CoV-2 infection was increased compared to those without infection by approximately 60%. Findings from meta-analyses were similar across different datasets in the US and Europe despite differences in criteria for defining incident diabetes, adjustment for covariates and duration of follow-up, and estimates were robust on sensitivity analyses. Although there was variability in reporting of subgroups among studies, overall diabetes risk was similar across different age groups [47.••••, 52], but potentially higher in males compared to females [45.••••], in patients with more severe disease [44.••••] and in the US compared to Europe [46.••••].

The findings of elevated risk of incident diabetes compared to non-COVID-19 respiratory illnesses suggest that SARS-CoV-2 increases risk beyond the general morbidity seen with viral illness. Further mechanistic studies are warranted to better understand the pathophysiology of diabetes associated with SARS-CoV-2 infection. Evidence is mixed regarding the association of SARS-CoV-2 infection with T1D. The role of autoimmunity, and validation studies in other populations, or using biochemical markers may be helpful in further elucidating the relationship. SARS-CoV-2 infection is associated with an elevated risk of T2D, but it is unclear of the timing of the incident diabetes and the persistence of hyperglycemia. Longitudinal studies are necessary to understand the prognosis of patients with incident COVID-19-associated diabetes with regard to their rates of remission, glycemic control, and need for glucose-lowering medications. Future studies should also evaluate the effect of factors that affect the severity of SARS-CoV-2 infection such as vaccination and viral variant, as one study suggested a protective effect for developing incident diabetes with vaccination [52]. It is also unclear what the role of socio-economic factors, such as lifestyle changes or healthcare utilization, have in the pathophysiology or detection patterns of disease. Analyses that incorporate social determinants of health and health care utilization behaviors are needed.

There are several limitations in the current literature. As most studies were conducted retrospectively using either electronic health records, claims or registry databases, ascertainment of diabetes status prior to SARS-CoV-2 infection and of COVID-19 diagnoses in patients who were either not tested or who tested at home, are limited. Additionally, as many studies relied on ICD-10 diagnosis codes, it is possible that diabetes cases were either missed or that the subtype was miscoded. Prospective studies in cohorts where ascertainment of diagnoses are possible would be helpful in validating these findings.

The current literature reports an increased risk of incident diabetes after SARS-CoV-2 infection. These findings may inform clinical practice regarding the need for increased monitoring for diabetes after acute SARS-CoV-2 infection in patients of all ages, and for counseling patients in preventive or therapeutic treatments that may mitigate the severity of illnesses such as vaccination or paxlovid. Further studies are needed to determine the mechanisms, mitigating factors and long-term outcomes of patients who develop incident diabetes after SARS-CoV-2 infection. Understanding the relationship of COVID-19 and incident diabetes during this pandemic may provide insight into strategies for understanding and mitigating the impact of future pandemics.
